# Encapsulation of Lactoferrin in Calcium-Alginate Microparticles and Its Release Therefrom Under Neutral and Mild Acidic Conditions: Synthesis, Characterization and Mathematical Modeling

**DOI:** 10.3390/gels11020116

**Published:** 2025-02-06

**Authors:** Teresa Paduano, Simona Zuppolini, Rosa Vitiello, Mauro Zarrelli, Riccardo Tesser, Anna Borriello

**Affiliations:** 1 Institute for Polymers, Composites and Biomaterials (IPCB)—National Research Council, 80055 Portici, Italy; teresapaduano@cnr.it (T.P.); mauro.zarrelli@cnr.it (M.Z.); anna.borriello@cnr.it (A.B.); 2 Department of Chemical Sciences, University of Naples Federico II, Complesso Universitario di Monte Sant’Angelo, 80126 Naples, Italy; riccardo.tesser@unina.it

**Keywords:** lactoferrin, alginate, microparticles, in vitro release, mathematical modeling, stimuli-responsive materials, biocompatibility

## Abstract

Bio-based polymeric stimuli-responsive materials have attracted increasing interest, especially in the pharmacological and nutraceutical fields. These materials mainly consist of macromolecules capable of conformational and chemical changes in response to external signals. One active molecule mostly used in bio-related areas is lactoferrin (Lf), which is attracting attention due to its beneficial effects (antimicrobial, anti-inflammatory, and anti-carcinogenic) on the human body. Since pH or temperature in the human body can promote Lf degradation, encapsulation in a suitable system is required. A valid solution is to encapsulate the Lf in a polysaccharidic matrix such as alginate (ALG) thanks to its biocompatibility and easy gelation with bivalent cations. This work aims to encapsulate iron-depleted Lf in alginate gel microspheres for stability improvement by ionic cross-linking with Ca^2+^ ions. The obtained particles were characterized in terms of structure, thermal stability, and morphology, and their swelling capability was determined. Release studies were carried out on the freeze-dried particles to investigate the effect of neutral pH 7 and acidic pH 5. At last, the optimization of the loaded system was completed by developing a mathematical model able to predict the swelling behavior of the carrier particle and the subsequent Lf kinetic release over time.

## 1. Introduction

Stimuli-responsive polymeric materials are in high demand in biomedical applications, pharmacology, and tissue engineering. External stimuli from the surrounding physiological environment (e.g., pH, light, temperature) can trigger chemical and/or conformational changes in the material. Subsequently, the release of encapsulated cargoes can be promoted [1,2]. One of the active molecules considered to be of great interest is lactoferrin (Lf) due to its attractive health benefits (i.e., antimicrobial, anti-inflammatory, immunomodulatory, and anti-carcinogenic effects) for humans and animals [3], which are attributed to its highly cationic nature [4]. Lf is a glycoprotein (MW ~ 80 kDa) able to bind to iron with high affinity [5], and for this, it exists in different forms: iron-depleted (apo-), iron-saturated (holo-), or as a mixture of apo- and holo-Lf (native). Lf plays an important role in the first line of the human defense system against microbial infections through two different mechanisms: bacteriostatic and bactericidal effects. In particular, the iron-depleted (apo-) form is considered responsible for the bacteriostatic effect in which bacteria are deprived of the iron necessary for cell growth [6], while the bactericidal effect is a type of membrane-mediated activity of negatively charged Lf that leads to cell death [7]. The use of this protein in industrial applications is limited due to the easy denaturation that would occur under processing conditions, including production, preservation, storage, and transportation [8]. Previous research has shown that even oral administration of Lf leads to reduced effects due to its breakdown under gastric conditions [9]. A valid strategy for optimizing the stability and safe delivery of Lf is encapsulating it into a biocompatible and stable matrix. For this purpose, alginate has been widely used for the encapsulation of Lf [10,11], and more generally, for the encapsulation of active molecules in drug delivery applications. Some existing methods for generating charged microparticles use complex techniques: lithography [12], bioprinting [13], or microfluidics [14]. The use of alginate makes the encapsulation process extremely simple thanks to its unique gelling properties in the presence of bivalent cations (e.g., Ca^2+^) [15]. This technique is useful for the encapsulation of heat-labile active agents as it does not require high temperatures. Sodium alginate is a natural polysaccharide extracted from brown algae (Phaeophyceae), and it is composed of sequences of β-D-mannuronic acid (M) and α-L-guluronic acid (G) residues of widely varying compositions and sequences. Carboxyl groups endow alginate with a high affinity for divalent and trivalent ions and cationic protein molecules [16]. Since Lf is a protein with positively charged regions, most evidently at the N-terminus, electrostatic interactions occurring with alginate ensure a higher stability of trapped proteins within the alginate gel matrix and minimize loss by diffusion [17,18]. Hence, a prolonged and controlled release of the protein from alginate particles can be allowed [19]. Alginate shows pH-responsive swelling behavior, and particles are used to control release in the oral delivery of drugs and proteins [20,21].

In this work, the release behavior of Lf-loaded alginate microparticles was investigated, exploring the effect of different surrounding pHs. The iron-depleted Lf form was chosen due to its interesting bactericidal effect. Furthermore, ad hoc-formulated mathematical models describing the swelling behavior and release kinetics of active ingredients from loaded systems were used to support the optimization of the release system. The created models will be based on systems of ordinary differential equations and adapted to different sets of experiments [22]. The novelty of this work is the development of a mathematical model that describes and can predict the phenomenon of the release of an active ingredient from charged microparticles in a very accurate and realistic way. The mathematical models currently present in the literature mainly describe phenomena of the adsorption of molecules from liquid or gas, glossing over those of release. These models consider only two phases (solid and liquid) and simple steps of capture (for adsorption) or release. Furthermore, they are empirical models that are based on the Lagergreen pseudo-first-order or pseudo-second-order model (which are all modifications of the model developed by Irving Langmuir for adsorption) [23,24].

## 2. Results and Discussion

### 2.1. Preparation of Microparticles

**Figure 1:**
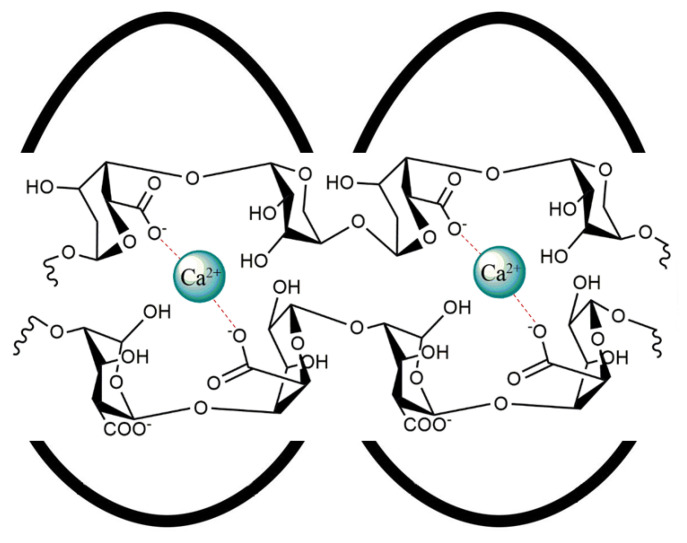
Polymer chain “egg-box” model.

The most common method used to prepare alginate microgels is ionic cross-linking in the presence of divalent cations, such as Ca^2+^. Alginate is formed by guluronate (G) blocks and mannuronate (M) blocks. In general, the gel structure is obtained thanks to the G blocks. The G blocks, having a high degree of coordination with the divalent ions, form junctions with the G blocks of the adjacent polymer chains in what is defined as the egg-box cross-linking model (Figure 1) [25].

### 2.2. Characterization of Microparticles

**Figure 2:**
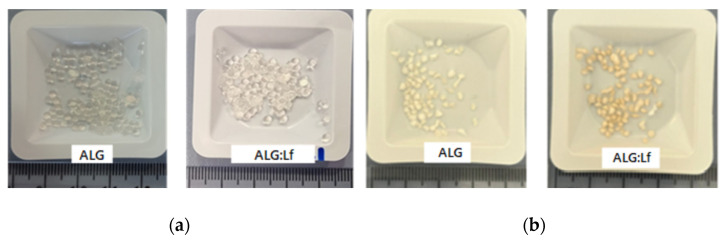
ALG and ALG:Lf microparticles in (**a**) swollen (after synthesis) and (**b**) freeze-dried form.

The Lf-loaded alginate microgels were prepared by exploiting the driving force of this mechanism, where the protein dissolved in solution was trapped into microgels during the cross-linking of polymer chains. The microgels obtained appear as transparent spheres with a homogeneous size distribution (~3 mm), and in the case of the ALG:Lf samples, a very light yellow color is observed due to the Lf content. This different coloration between neat and loaded samples is more evident after freeze-drying microgels (Figure 2).

**Figure 3:**
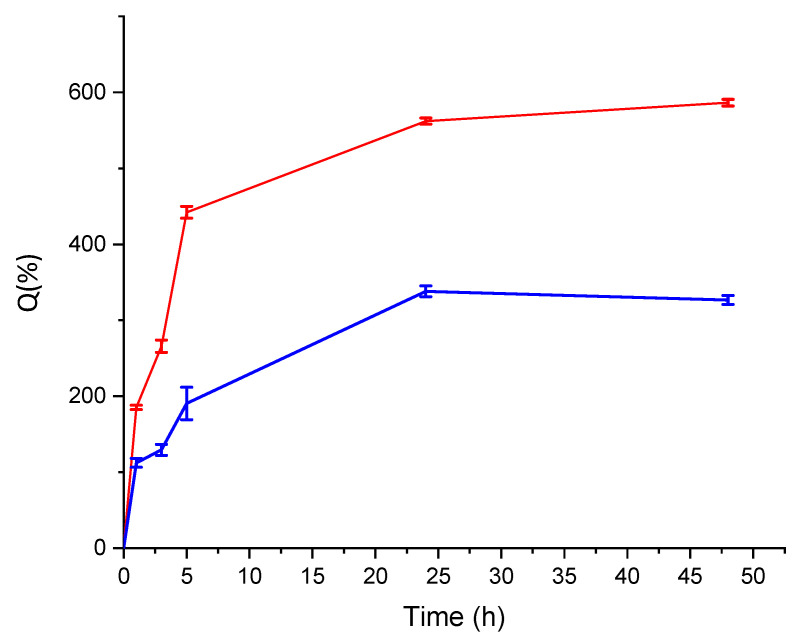
Swelling degree of (Q (%)) microspheres: interval plots of ALG (red curve) and ALG:Lf (blue curve) particles.

The results obtained from the swelling experiments on freeze-dried microparticles are reported in Table 1. A similar behavior can be observed for both ALG and ALG:Lf samples: the microparticles swell very quickly in the initial hours and reach their maximum swelling volume between 24 and 48 h. The graphs of swelling degree (Q (%)) versus time (Figure 3) clearly show that the swelling process of the microspheres was faster for the neat ALG microparticles and slower for the loaded microparticles. The swelling degree of the freeze-dried microspheres depends on the presence of Lf; the ALG:Lf microparticles have lower water retention ability due to steric protein hindrance, which allows for poorer water adsorption. Firstly, the freeze-dried ALG:Lf particles swell, and then the dynamic release of the protein is triggered thanks to the establishment of an Lf concentration difference between the internal environment and the external bulk solution. This results in a weight and dimension loss in the swollen particles due to the higher specific weight of the protein.

The sizes of the neat and Lf-encapsulated particles were measured using ImageJ 1.54g software, and they are expressed as a weighted average of the diameter (Table 2). The particle sizes of the freshly synthesized swollen microgel particles (ALG and ALG:Lf) were not significantly different from those of the freeze-dried ones, which were rehydrated (r-ALG and r-ALG:Lf), losing only 6–7% of their swelling power and recovering their original shape (Table 2).

This behavior suggests that the freeze-drying process does not induce the collapse of the inner structure of primary microgel particles, and the shape of the particles is preserved during rehydration [26]. Regarding the size, the results indicate that the ALG particles are bigger than the Lf-loaded ones, including both the freshly swollen and rehydrated particles. This is probably due to the presence of lactoferrin, which affects the water capture ability of the particles.

**Figure 4:**
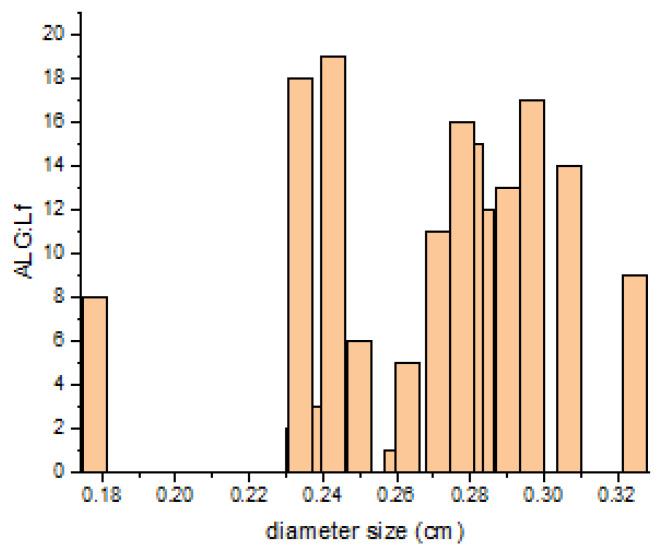
Size distribution of swollen ALG and ALG:Lf particles.

Figure 4 shows the size distribution of swollen ALG and ALG:Lf particles.

**Figure 5:**
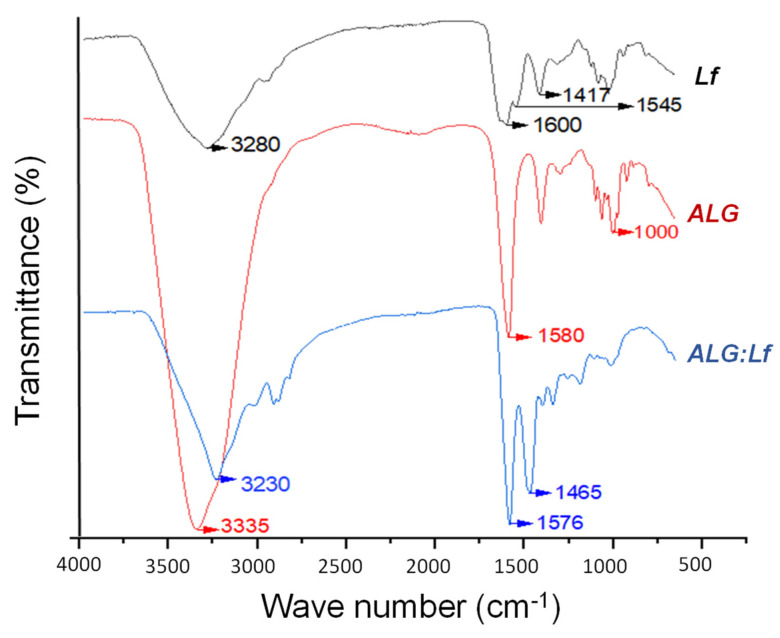
FTIR-ATR spectra of Lf, ALG, and ALG:Lf microparticles.

FT-IR spectra are reported in Figure 5. In the ALG spectrum, the vibrational stretching of O–H bonds is observable in the range of 3600–3000 cm^−1^, centered at 3335 cm^−1^. Furthermore, the characteristic peaks of the stretching of the carboxyl (-COOH) and ether (C–O–C) groups are observable at 1580 and 1000 cm^−1^, respectively. The Lf spectrum shows the characteristic amide bands I, II, and III observed at 1600, 1545, and 1417 cm^−1^, respectively. Furthermore, free -OH groups of amino acids can be identified from the band at 3280 cm^−1^ [27]. The spectrum of ALG:Lf shows the signals of both polymers, particularly the bands at 3230, 1576, and 1465 cm^−1^, attributable, respectively, to the free O-H groups of the amino acids [28], the stretching of the N-H group, and the stretching of the C-N and N-H groups. The displacement of 4 cm^−1^ at 1576 cm^−1^ (amide I) is noteworthy compared to the value for lactoferrin, which can be attributed to the association between the negatively charged groups (-COO^−^) of alginate and the positive ones (-NH_3_^+^) of Lf [29].

**Figure 6:**
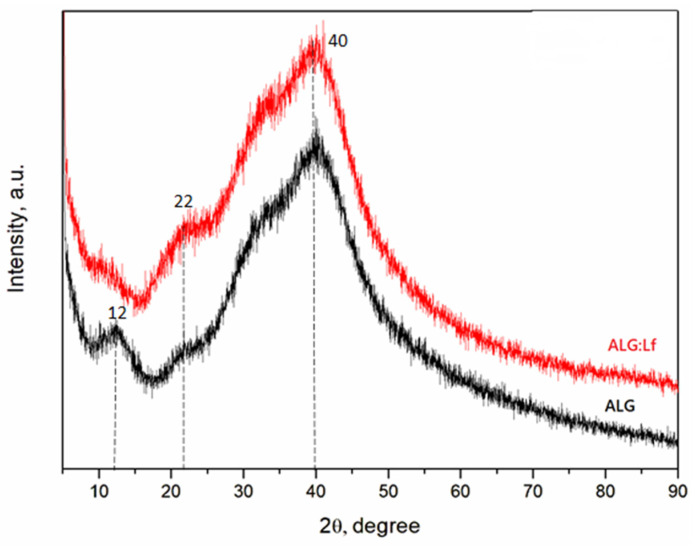
XRD pattern of calcium-alginate beads (ALG, black curve) and Lf-loaded calcium-alginate beads (ALG:Lf, red curve).

The possible effect of Lf entrapment on the physical state of the calcium-alginate (ALG) matrix was investigated by X-ray diffraction (XRD) analysis (Figure 6).

In the XRD diffractogram of the ALG particles (black curve), the presence of main crystalline peaks at 2θ diffraction angles of 12°, 22°, and 40° confirms its characteristic semicrystalline nature [30]. For the ALG:Lf particles (red curve), while the third peak remains stable at 40°, the first peak disappears, indicating a decrease in crystallinity. These findings suggest that the presence of the protein (80 kDa) and its intermolecular interaction with crosslinked alginate chains can cause internal deformation through the fracture of the alginate grains into subgranules during the preparation of Lf-loaded beads interfering with the semicrystallinity order of the alginate [30].

**Figure 7:**
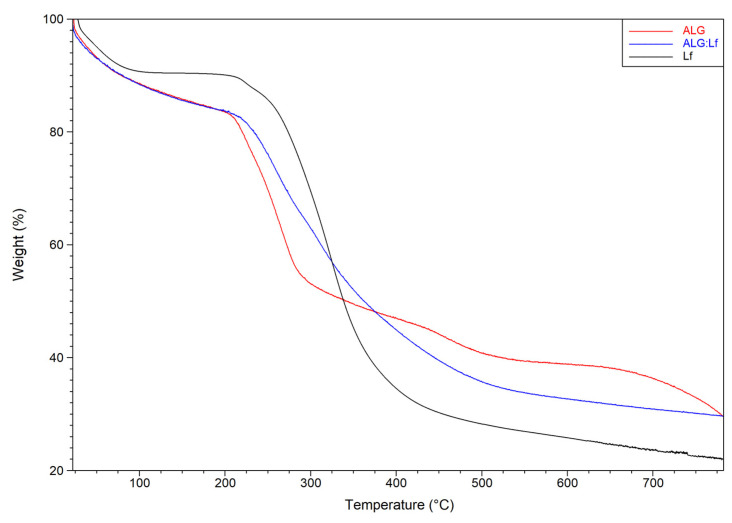
TGA curves of Lf, ALG, and ALG:Lf microparticles.

The thermogravimetric curves obtained for the ALG and ALG:Lf microparticles (Figure 7) show the presence of three degradation mechanisms: (I) The first is between 30 °C and 210 °C, where a minimal initial weight loss in alginate is observed due to dehydration, followed by rapid degradation to CaCO_3_ with a thermal degradation midpoint at 197 °C. In the second temperature range II (210–300 °C), the percentage weight loss can be attributed to the breaking of the glycosidic bonds in the alginate backbone (T_max_ = 245.6 °C) and the consequent loss of its abundant hydroxyl groups in the form of water. The third mechanism (III), in the highest temperature range (350–500 °C), is attributable to the material’s decarboxylation with the formation of calcium oxide and hydroxide (up to 700 °C), leading to a final weight residue of about 30%. For the ALG:Lf microparticles, a higher degradation temperature (~275 °C) was observed, suggesting a stability increase attributable to the presence of the Lf protein, whose thermogram (black curve) shows the beginning of degradation at 210 °C, which lasts until a final residue of about 20% is obtained.

**Figure 8:**
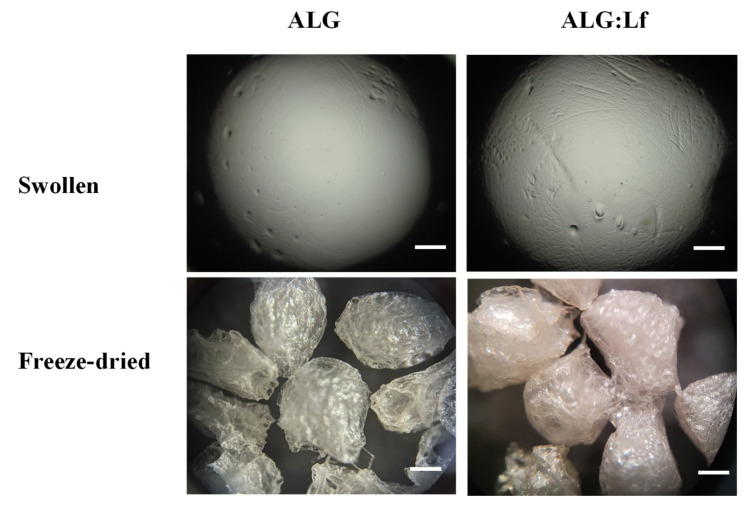
Optical microscopy images of ALG and ALG:Lf microparticles at 200 µm of magnification.

The morphological analysis of the ALG and ALG:Lf microparticles was preliminarily performed by optical microscopy observations for both the swollen and freeze-dried samples, and the images are shown in Figure 8. For both swollen microgels, no significant differences in size (about 3 mm) were observed, aside from the rougher surface of the microgels. In the case of the freeze-dried particles (size of about 2 mm), a different color was observed; the ALG:Lf particles appear more consistent and orange-colored than the colorless ALG ones.

**Figure 9:**
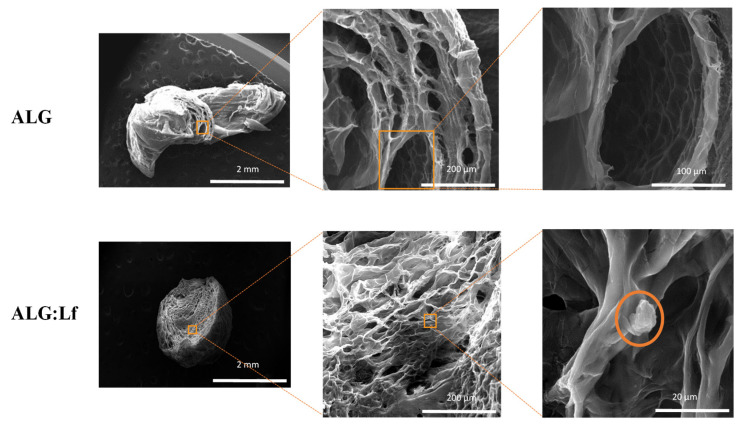
SEM micrographs of ALG and ALG:Lf swollen microparticles. Internal section analysis at different magnifications (2 mm, 200 µm, 100 µm, 20 µm). In the bottom right figure, Lf agglomerates are highlighted by the orange.

The morphological analysis of the microparticles was completed by SEM observations. To observe the internal microgel structure, the swollen ALG and ALG:Lf samples were pre-treated with liquid nitrogen and crushed. The SEM images (Figure 9) show the presence of a porous structure for both samples, and the presence of protein agglomerates (orange circled) in the ALG:Lf microparticles is evident.

**Figure 10:**
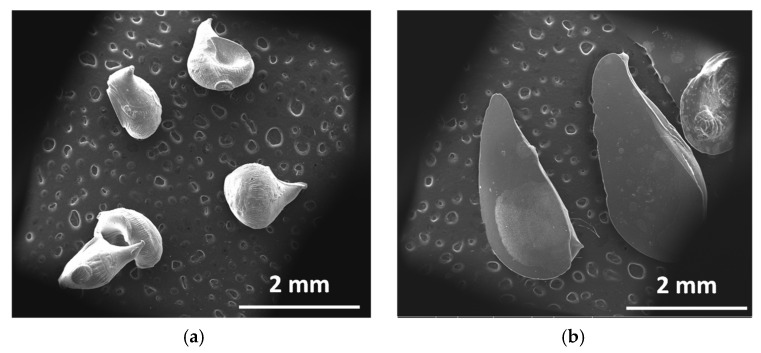
SEM micrographs of (**a**) ALG and (**b**) ALG:Lf freeze-dried microparticles.

In the SEM images of the freeze-dried particles (Figure 10), a difference in the particles’ shape was observed: the ALG samples show a round shape, while the Lf-loaded ones have a more elongated shape, probably due to the presence of the encapsulated protein.

The encapsulation efficiency (EE (%)) of Lf was determined for five different batches of particles, and the results are reported in Table 3. The results estimated an encapsulation efficiency for the encapsulation of Lf into ALG microgels equal to 65.7% (SD ± 8.3).

#### 2.2.1. 2D-PAGE Analysis

The results obtained from the 2D-PAGE analysis for iron-depleted bovine (apo-) lactoferrin (total protein 95%) estimated an isoelectric point (pI) of 8.0–8.4, which is in line with the range of 8.0–9.0 reported in the literature [18]. It is worth noting that 2D-PAGE analysis is considered a sophisticated analytical method, obtained by combining two techniques:

- Isoelectric focusing (IEF) is based on the separation of proteins according to their different isoelectric points. The separation occurs by applying a potential difference at the end of the gel containing a pH gradient. The gradient is formed by introducing ampholyte molecules into the gel.
- Separation of proteins based on molecular weight (SDS-PAGE).

#### 2.2.2. In Vitro Release Studies

**Figure 11:**
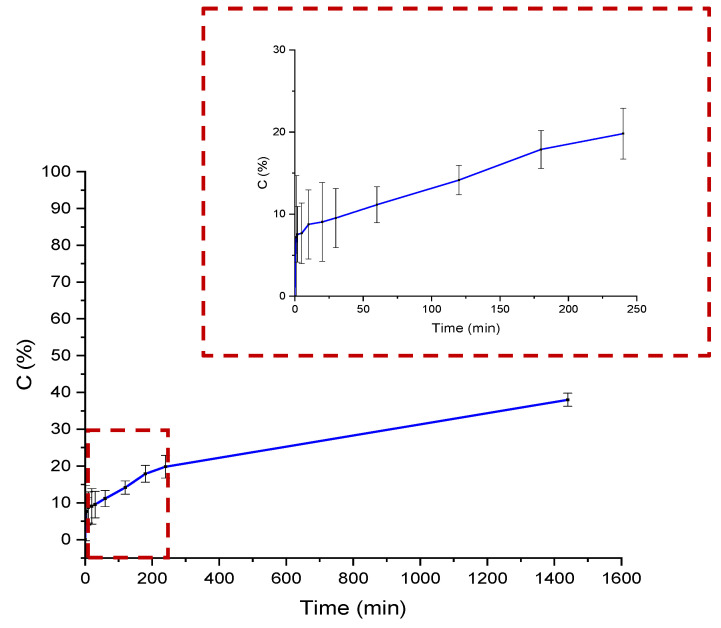
In vitro Lf release profile from loaded ALG particles at neutral pH.

The release of bioactive encapsulated compounds is regulated by the diffusion or dissolution of the gel particles or a combination of both phenomena [31]. When ALG:Lf microparticles come into contact with the aqueous solution, the solvent penetrates the porous alginate structure, allowing polysaccaridic material to swell to its maximum size. This behavior promotes the diffusion process of the encapsulated Lf outside of the matrix and, consequently, its release. To evaluate the effect of pH on the Lf release, experiments were performed at neutral pH and pH 5. It is worth noting that a weakly acidic environment was recognized to mitigate Lf denaturation, which can be promoted by a lower pH, while a neutral pH is guaranteed to work below the pH of Lf (8–8.4). In vitro release tests on Lf-loaded microparticles were performed on the freeze-dried samples, as these represent a more interesting system due to the higher stability of the encapsulated protein and the possibility of constituting a useful and practical platform for delivery in nutraceutical applications. Figure 11 shows the release profile of Lf as the cumulative protein concentration (%) over time at pH 7. The results show that 40% of trapped Lf was detected within 24 h, revealing the slow release of the protein under neutral conditions.

**Figure 12:**
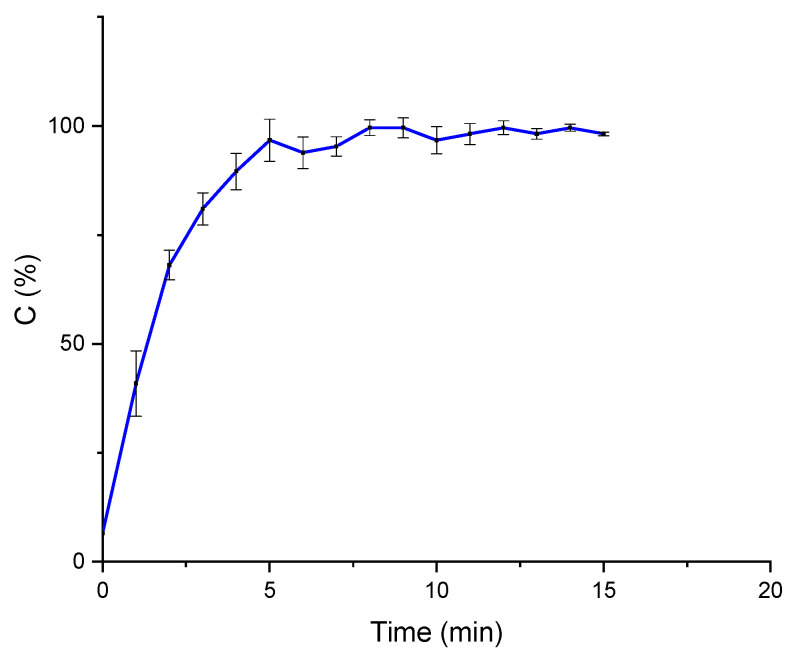
In vitro Lf release profile from loaded ALG particles at pH 5.

On the other hand, an acidic pH seems to have a significant impact on both the kinetics and the amount of Lf released, as shown in Figure 12. In fact, at pH = 5, almost 90% of the incorporated Lf is released within a few minutes (6 min), showing faster release kinetics due to the dissolution of the particles [32].

### 2.3. Mathematical Models

This work focuses on developing and validating mathematical models describing the release behavior of active ingredients from loaded microparticles. Modeling was performed via MATLAB R2024a, a well-known software tool for scientific numerical calculations and statistical analysis. The release of encapsulated active molecules occurs through two simultaneous phenomena:

- The microparticles swell when in contact with an aqueous solution (external bulk), penetrating the porous structure of the freeze-dried particles.
- As the swelling proceeds, the active substance migrates by diffusion from the polymer structure into the adsorbed liquid phase, and, from this, it moves towards the liquid bulk solution. The driving force is then the difference in concentration between the adsorbed liquid phase and the external bulk of the liquid. This driving force is initially very high, and the corresponding release phenomenon is quite fast. Over time, the release rate diminishes, and a concentration plateau is reached.

To develop a model for encapsulated molecule release, both the dissolution (during swelling) and diffusion of molecules through the polymer matrix to the external bulk fluid were considered. In the case of the studied alginate-based system, two mathematical models were developed to describe these phenomena for Lf release at acidic pH: the “Swelling model” and the “Dynamic release model”.

Mathematical modeling was performed to describe the release phenomenon at pH 5; this is because acidic pH is a potential environmental pH of interest for numerous applications.

The models led to a system of ordinary differential equations, and the obtained models were then fitted to different experimental datasets with satisfactory results, indicating that the models presented here, despite their simplicity, can describe swelling and release phenomena.

#### 2.3.1. Swelling Model

The swelling model is needed to understand how particles swell over time from the calculation of the swelling constant (k_v_). At zero time (t = 0), the particle is considered a dry solid (V_ads_ = 0). The liquid’s penetration into the dry particle occurs with swelling, which decreases over time. Moreover, the acidic environment used led to the polymer chains opening with the dissolution of the microparticle’s external shell. The aqueous solution penetrates the particle, which swells up to a maximum swelling volume (V_max_). The swelling rate is a function of time, and it linearly depends on the swelling constant (k_v_) and on the volume difference between the maximum swelling volume and the variable adsorbed volume over time. The swelling measurements are given by the ALG:Lf sample particles at various times: 0, 1 min, 3 min, 5 min, 24 h, and 48 h. The adsorbed volume change over time is expressed by the following differential equation:

(1) $$\frac{{\mathrm{dV}}_{\mathrm{ads}}}{\mathrm{dt}}=k_{v}\;\left(V_{\max}-V_{\mathrm{ads}}\left(t\right)\right)$$

When the particle volume reaches its maximum value, no more volume increase is expected. The constant k_v_ can be determined through the fitting of experimental data related to the adsorbed volume as a function of time [33].

#### 2.3.2. Dynamic Release Model

**Figure 13:**
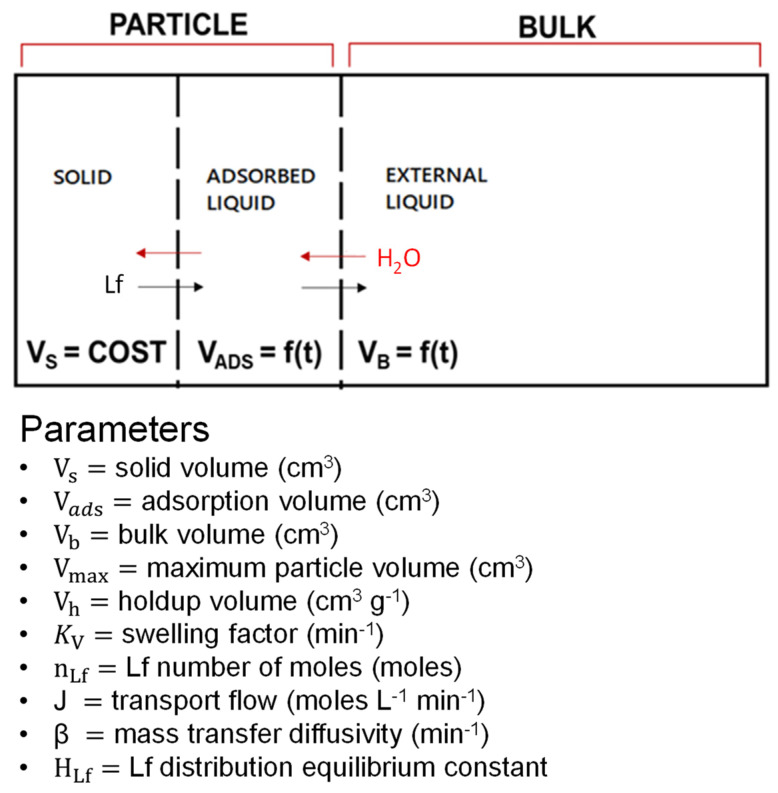
Dynamic release model.

Polymer microparticles are modeled as a perfect sphere with a variable volume, whose increase is due to the swelling model previously described. This assumption represents one of the limitations of the model. The calculations were performed assuming that all particles have the same size (uniform distribution), whose value is equal to their average diameters. This consideration assumes that the swelling phenomenon is the same for all particles, but this consideration is probably not true, as in reality, there is a distribution of diameters. At zero time, the particle is considered a dry solid (V_ads_ = 0), with an initial volume (V_s_) and weight (W_s_). The following diagram describes the dynamic release model (Figure 13).

This model assumes that the bulk solution penetrates the microparticles and swells them; simultaneously, the Lf inside the particle matrix diffuses outside towards the external bulk solution. In the described mathematical model, two possible limiting effects are considered. The first one is due to the stagnant liquid layer adherent to the solid inside the microparticle, described by the solid–liquid (liquid-side) mass transfer coefficient (β_sl_). The second one is due to the resistance to mass transfer located at the two-liquid interface (adsorbed liquid and external liquid). A rigorous treatment would require a two-film approach [34], but, in our case, we have simplified this aspect by using single-film resistance by using a liquid–liquid global mass transfer coefficient (β_ll_).

The particle volume changes over time are described by the following volume balance equations:

Volume balance:

(2) $$\frac{{\mathrm{dV}}_{\mathrm{ads}}}{\mathrm{dt}}=+{\;k}_{v}\left(V_{\max}-V_{\mathrm{ads}}\right)$$

(3) $$\frac{{\mathrm{dV}}_{b}}{\mathrm{dt}}=-{\;k}_{v}\;\left(V_{\max}-V_{\mathrm{ads}}\right)$$

The release occurs first in the internal core particle due to the adsorbed volume solution (V_ads_), which interacts with the Lf. In this way, inside the particle, it creates a concentration gradient that spreads Lf towards the external bulk volume (V_b_) until equilibrium is reached or the particle dissolves (due to external factors such as the acid pH of the bulk solution). Polymer degradation occurs due to the hydrolysis mechanism: the aqueous solution penetrates the matrix, breaking the ester bonds, which constitute the polymer backbone. After a long time, in some cases, the particles dissolve completely, and there is no more notable release.

The two limiting phenomena that occur during Lf diffusion, described by the solid–liquid mass transfer coefficient (β_sl_) and by the liquid–liquid mass transfer coefficient (β_ll_), are needed to calculate the transport flows. J_sl_ and J_ll_ are the transport flows through the solid and liquid phases, which depend on the difference between the Lf concentration adsorbed and the Lf concentration in bulk. In particular, J_sl_ depends on the Lf distribution equilibrium constant between the solid and liquid phases (H_Lf_). The constant H_Lf_ represents the Lf distribution between the solid and liquid phases. It is defined as the ratio given in Equation (4):

(4) $$H_{\mathrm{Lf}}=\frac{C_{\mathrm{sLf}}}{C_{\mathrm{adsLf}}\;}\;$$

The transport flows are described by the following balance equations:

(5) $$J_{\mathrm{sl}}=\beta_{\mathrm{sl}}\;\left(\frac{C_{\mathrm{sLf}}}{H_{\mathrm{Lf}}}-C_{\mathrm{adsLf}}\right)$$

(6) $$J_{\mathrm{ll}}=\beta_{\mathrm{ll}}\;\left(C_{\mathrm{adsLf}}-C_{\mathrm{bLf}}\right)$$

At zero time, the particles contain several Lf moles (n_Lfs0_). The Lf moles are a time function and depend linearly on the transport flow between the phases. The mass balance of the moles shows that the mass of Lf moles that exit the solid particle must be the same as the mass of moles that will be found in the bulk solution.

Mole balance equation:

(7) $$\frac{{\mathrm{dn}}_{\mathrm{sLf}\;}}{\mathrm{dt}}=-J_{\mathrm{sl}}{\;V}_{\mathrm{ads}}$$

(8) $$\frac{{\mathrm{dn}}_{\mathrm{adsLf}\;}}{\mathrm{dt}}=+J_{\mathrm{ll}}{\;V}_{\mathrm{ads}}-J_{\mathrm{sl}}{\;V}_{\mathrm{ads}}$$

(9) $$\frac{{\mathrm{dn}}_{\mathrm{bLf}\;}}{\mathrm{dt}}=+J_{\mathrm{ll}}{\;V}_{\mathrm{ads}}$$

All the considered mass balances are valid until the particle dissolves; after its eventual dissolution, all the charged lactoferrin will end up in the bulk solution.

**Figure 14:**
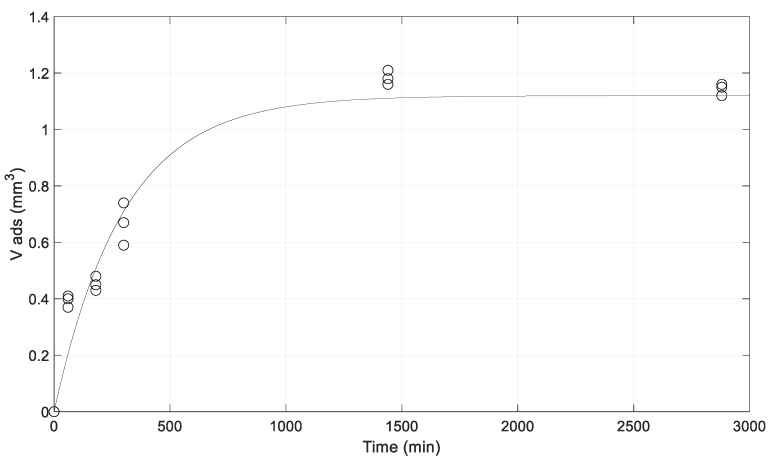
Logarithmic graph of the swelling of ALG:Lf freeze-dried particles.

The measurement of swelling volumes was performed using the weight data of the particles obtained during the swelling process, as reported in paragraph 2.2. Table 4 shows the values of the particle weights over time, and these are plotted in Figure 14. All the experiments were carried out in triplicate, and the results are presented as the mean ± SD.

Matlab Optimization Toolbox allows one to find parameters that minimize or maximize the objectives while satisfying the constraints. The solvers in this toolbox can perform optimization tasks, such as k_v_ parameter estimation. The optimized k_v_ value obtained from MATLAB is k_v_ = 0.00335 min^−1^.

**Figure 15:**
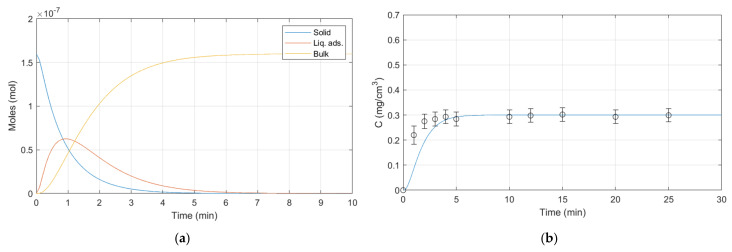
Batch 1 dynamic model release: (**a**) Lf moles vs. time; (**b**) Lf concentration vs. time.

**Figure 16:**
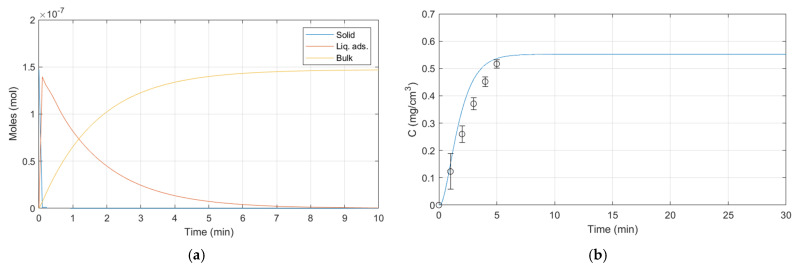
Batch 2 dynamic model release: (**a**) Lf moles vs. time; (**b**) Lf concentration vs. time.

**Figure 17:**
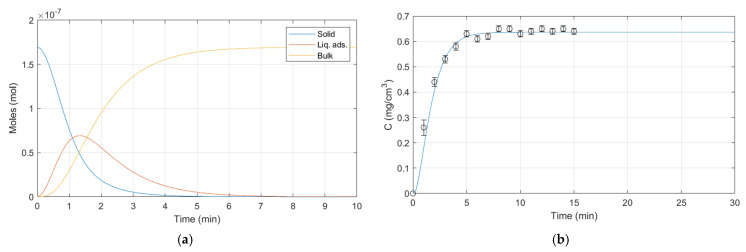
Batch 3 dynamic model release: (**a**) Lf moles vs. time; (**b**) Lf concentration vs. time.

The simulations performed via MATLAB reflect the operating conditions used for releases. Some experimental parameters are fixed and others are varied to verify the validity of the dynamic release model for ALG:Lf particles under different conditions. The room temperature (23 ± 2 °C) and the external bulk pH (citric acid/sodium hydroxide buffer solution, pH 5) are fixed parameters, while the external bulk volume and the presence of stirring are varied. Three different batches of ALG:Lf particles were considered to perform experimental releases over time:

- Batch 1: 40 mL of external bulk kept under stirring (Figure 15);
- Batch 2: 20 mL of external bulk kept under stirring (Figure 16);
- Batch 3: 20 mL of stagnant external bulk (Figure 17).

The graphs below show the evolution of lactoferrin release over time until equilibrium is reached or the particle is dissolved (the particle is dissolved in the release graphs at the point shown by the red dotted line). The release data are given as an average of three measurements.

For BATCH 1, the standard deviation was calculated (SD = ±0.008) and assumed to be the same for the release tests of BATCH 2 and BATCH 3.

The error bars were calculated as the ratio between the error (standard deviation) and the concentration, since the uncertainty on the measurement is higher at low concentrations.

Batch 1 parameters:

W_s_ = 0.025 (g);

V_s_ = 0.00024 (cm^3^);

V_ads0_ = 0.00000001 (cm^3^);

V_h_ = 3.82 (cm^3^/g);

V_b0_ = 40 (cm^3^);

K_v_ = 0.00335 (min^−1^);

n_Lfs0_ = 0.012/75,000 (moles);

n_Lfads0_ = 0 (moles);

n_Lfb0_ = 0 (moles);

H_Lf_ = 0.00013 (-);

β_sl_ = 2889.62500 (min^−1^);

β_ll_ = 1.01277 (min^−1^).

Batch 2 parameters:

W_s_ = 0.023 (g);

V_s_ = 0.00024 (cm^3^);

V_ads0_ = 0.00000001 (cm^3^);

V_h_ = 3.82 (cm^3^/g);

V_b0_ = 20 (cm^3^);

K_v_ = 0.00335 (min^−1^);

n_Lfs0_ = 0.012/75,000 (moles);

n_Lfads0_ = 0 (moles);

n_Lfb0_ = 0 (moles);

H_Lf_ = 0.00015 (-);

β_sl_ = 2997.62500 (min^−1^);

β_ll_ = 0.60261 (min^−1^).

Batch 3 parameters:

W_s_ = 0.024 (g);

V_s_ = 0.00024 (cm^3^);

V_ads0_ = 0.00000001 (cm^3^);

V_h_ = 3.82 (cm^3^/g);

V_b0_ = 20 (cm^3^);

K_v_ = 0.00335 (min^−1^);

n_Lfs0_ = 0.013/75,000 (moles);

n_Lfads0_ = 0 (moles);

n_Lfb0_ = 0 (moles);

H_Lf_ = 0.00016 (-);

β_sl_ = 41.43750 (min^−1^);

β_ll_ = 0.48151 (min^−1^).

The mathematical models currently present in the literature describe adsorption and release phenomena in a very simplified way and based on empirical models. Therefore, in the present work, a more sophisticated mathematical model was developed that can describe and predict the release phenomenon of an active ingredient from charged microparticles in a very accurate and realistic way. This developed model supports the study of release carried out at pH 5 from charged particles, but it is valid for any other pH value of the bulk solution in which the release is carried out. In this mathematical modeling, the ALG and ALG:Lf microparticles, which in fact have a porous internal structure, are compared to sponges (as can be seen from the SEM images Figure 8). This modeling, unlike the others present in the literature, considers two simultaneous phenomena: the swelling of the particle and the release of the active ingredient, correlated to each other by the swelling constant k_v_.

Furthermore, the model reflects three steps:

(1) The system captures water and swells;
(2) Lactoferrin migrates from the internal solid to the adsorbed liquid;
(3) Lactoferrin migrates to the external mass.

The solid–liquid (β_sl_) and liquid–liquid (β_ll_) mass transfer coefficients and the solid–liquid equilibrium constant (H_Lf_) are the three parameters estimated for the fitting of the experimental data. For data validation, the estimated parameters for one BATCH (e.g., BATCH 1: β_sl_, β_ll_, H_Lf_) were used to describe other experimental data (e.g., BATCH 2 and BATCH 3). This attempt led to satisfactory results. For all the systems examined, the constant H_Lf_ fluctuates around a value of the order of 10^−5^. This means that in equilibrium conditions and in close proximity to the film, the concentration in the adsorbed liquid is very high, and this creates a transport gradient from the solid to the adsorbed liquid. The values obtained from Matlab data fitting describe the experimental data for Lf release from the freeze-dried ALG:Lf particles, with a certain accuracy for all three BATCHES considered. In particular, the variation in β_ll_ influences the fitting data, so β_ll_ is considered a limiting parameter. The developed model is just a bit sensitive to the parameter H_Lf_ and not very sensitive β_sl_, which does not influence the fitting data. Varying the value of β_sl_ does not lead to a variation in the fitting data.

## 3. Conclusions

In this work, lactoferrin-loaded alginate microparticles were prepared in a one-step ionic cross-linking procedure and characterized. The in vitro release behavior of the freeze-dried system was investigated, such as the effect of pH on the kinetic release profile. The results showed that a weakly acidic environment (pH = 5) promotes the release of a higher amount of Lf (about 90%) in a shorter time compared to neutral pH conditions, in which only 40% of the protein was released.

The mathematical models developed support the study of Lf release at pH 5 but can be extended to different pH environments. The novelty of this work was the opportunity to develop a mathematical model to describe and predict the phenomenon of the release of an active ingredient loaded into microparticles in a very accurate and realistic manner. These models were based on systems of ordinary differential equations and were adapted to different sets of experimental data with satisfactory results.

The findings of this work highlight the potential of mathematical models to act as a supporting tool for the design of an ad hoc stimuli-responsive carrier system by optimizing experimental work.

## 4. Materials and Methods

### 4.1. Materials

Sodium alginate (Manugel GHB, average MW 97 kDa, M/G ratio 0.59, FMC BioPolymers UK Ltd. Girvan, Ayrshire, KA269JN, UK), bovine iron-depleted (apo-)lactoferrin (total protein 95%, Fonterra, Auckland, New Zealand), and anhydrous calcium chloride (CaCl_2_) (Sigma Aldrich, Milan, Italy) were used as received for the preparation of microgels. Tris(hydroxymethyl)aminomethane hydrochloride (Tris-HCl, Sigma Aldrich, Milan, Italy) and citric acid/sodium hydroxide (pH 5, VWR, Milan, Italy) buffer solutions were used for the kinetic release study.

### 4.2. Preparation of Microparticles

For the preparation of alginate microgels, 3 mL of sodium alginate solution (2 wt%) was added dropwise to 18 mL of 0.1 M CaCl_2_ solution under continuous stirring. The ionic cross-linking reaction of the alginate by Ca^2+^ occurred within 15 min. The alginate microgels (ALGs) formed and suspended in solution were collected and washed three times with Millipore water. For the preparation of Lf-loaded microgels (ALG:Lf), the same procedure was followed, starting from a solution of both components prepared by mixing equal volumes of 2 wt% stock solutions in a 1:1 ratio and stirring for about 10 min until a uniform mixture was obtained. The ALG:Lf microgels obtained were collected and washed three times with Millipore water and freeze-dried.

### 4.3. Characterization of Microparticles

#### 4.3.1. Structural Analysis

Attenuated total reflectance–Fourier transform infrared (ATR-FTIR) and X-ray diffraction (XRD) spectra were recorded for the structural analysis of the freeze-dried samples. The ATR-FTIR analysis was carried out on a Bruker Vertex V70 (Milan, Italy) in the range of 4000–400 cm^−1^ with a resolution of 4 cm^−1^ and 32 scans. The X-ray diffraction pattern was performed in an X-ray diffractometer (XRD), Bruker Phaser D2 (2nd generation). The lamp was a Cu tube with a monochromatic radiation wavelength (k = 1.54 Å). The working current was set as 30 mA, and the voltage was set as 40 kV. The data were obtained in a 2θ versus intensity (a.u) plot, scanning over a 2θ range from 2 to 90°.

#### 4.3.2. Morphological Analysis

The morphological analysis of the ALG and ALG:Lf microparticles was performed by optical microscopy (using the Optical Olimpus BX51 Instrument) at different magnifications and completed by Electron Microscopy (SEM) using an FEI Quanta 200 FEG equipment (Hillsboro, OR, USA) under high vacuum conditions, using a voltage of 20 kV. Samples were previously metalized through the deposition of a 5/10 nm thick Au/Pd layer using an Emitech K575X sputter coater (Quorum Technologies Ltd., Kent, UK). The swollen microgels were pre-treated with liquid nitrogen and crushed to observe the internal section at low vacuum pressure and low voltage.

#### 4.3.3. Thermal Analysis

Thermogravimetric analysis (TGA) was performed using a Netzsch TG 29 F1 Libra system (Netzsch Geratebau GmbH, Selb, Germany) under N_2_ atmosphere (50 mL/min) and at a heating ramp of 10 °C/min, within the temperature range 25–800 °C. Sample weights of around 6 ± 0.5 mg were used for the run tests.

#### 4.3.4. Swelling Degree Study

The swelling experiments were performed on freeze-dried ALG and ALG:Lf microparticles. In a typical test, the sample was immersed in 3 mL of Millipore water. At different time intervals (4, 24 and 48 h), the sample was extracted, and its weight was carefully determined after removing excess water from the surface by wiping with Whatman filter paper (Sigma Aldrich, Milan, Italy) [35]. Q (%) was calculated using Equation (10).

(10) $$Q\left(\%\right)=\frac{M_{\mathrm{sw}}-{\;M}_{d}}{M_{d}}\times 100$$

where M_sw_ is the mass of swollen microspheres and M_d_ is the mass of dry microspheres.

All the experiments were carried out in triplicate, and the results are presented as the mean ± SD.

#### 4.3.5. Particle Size Measurement

Swollen and freeze-dried microparticle sizes were processed using ImageJ 1.58g software. The image analysis was based on the calculation of the pixel value area in a user-defined section. Freshly prepared swollen microgels were collected and filtered after washing with Millipore water. All measurements were performed at room temperature (23 ± 2 °C) on an average of three samples.

#### 4.3.6. 2D-PAGE Analysis

The powdered Lf sample was incubated at 43 °C for 0, 2, and 4 h before 2D-PAGE analysis. After this, 25 μg and 50 μg of Lf were loaded, respectively, onto immobilized pH gradient gel (IPG) strips (pH 7–10 and pH 3–10, 7 cm; IPG ReadyStrip™ strips, Bio-Rad Laboratories, Hercules, CA, USA) that had been rehydrated for 12 h before use in a solution containing 8 M urea, 4% CHAPS, 100 mM DTT, 0.2% Bio-Lytes (IPG ampholytes), and 0.001% bromophenol blue. The detailed procedure is reported in ref. [36].

#### 4.3.7. In Vitro Release Tests

UV absorption measurements were performed using an Agilent Technologies Cary 60 UV–Vis spectrophotometer (Santa Clara, United States). A linear calibration curve for Lf (0.05–1.0 mg/mL) was obtained at λ_max_ = 280 nm. A typical in vitro release test of Lf loaded into freeze-dried ALG microparticles was carried out by placing the sample in a definite volume (40 mL) of releasing medium at room temperature under constant weak stirring. At predetermined time intervals, 3.0 mL of solution was taken out for analysis, and the analyzed aliquot was added back to the stirring system to maintain a constant volume. All the experiments were conducted three times to confirm reproducibility. The cumulative release of Lf (C%) versus time was calculated.

#### 4.3.8. Encapsulation of Lf

To determine the amount of Lf encapsulated inside the particles and the loading capacity, the reaction bulk (CaCl_2_ solution) and the obtained washes were analyzed at λ_max_ = 280 nm, using the following equations reported elsewhere [37].

EE (%) was calculated by the following Equation (11):

(11) $$\mathrm{EE}\left(\%\right)=\frac{m-\mathrm{mf}}{m}\times 100$$

where m is the total Lf amount, and mf is the free amount of Lf calculated at zero time.

## Figures and Tables

**Table 1:** Swelling degree Q (%) from weight of microspheres.

Sample	Q (%)				
	1 h	3 h	5 h	24 h	48 h
ALG	185.3 ± 2.8	265.1± 8.2	442.1 ± 7.8	562.0 ± 4.1	586.4 ± 4.5
ALG:Lf	112.4 ± 5.9	129.5 ± 7.2	190.5 ± 21.4	338.1 ± 7.2	326.7 ± 5.9

**Table 2:** Size measurement of (freshly synthesized) swollen and rehydrated microparticles.

Sample	Average Diameter (cm)
ALG	0.29 ± 0.03
ALG:Lf	0.27 ± 0.03
r-ALG	0.26 ± 0.02
r-ALG:Lf	0.21 ± 0.01

**Table 3:** EE (%) of Lf in ALG microgels.

Sample	EE (%)
B1	55.4
B2	62.6
B3	78.1
B4	67.9
B5	64.8

**Table 4:** ALG:Lf microparticles’ weight measurement over time during the swelling process.

Sample	Time (h)	Weights (mg)
ALG:Lf	0	0.35 ± 0.01
	1	0.75 ± 0.02
	3	0.80 ± 0.07
	5	1.08 ± 0.02
	24	1.60 ± 0.02
	48	1.62 ± 0.02

## Data Availability

The original contributions presented in this study are included in the article. Further inquiries can be directed to the corresponding authors.
